# Coloring complex shapes decreases patient anxiety in three care environments: a pilot study with color analysis

**DOI:** 10.3389/fpsyg.2024.1336202

**Published:** 2024-02-21

**Authors:** Manuel Dias Alves, Elodie Olmi, Jean-Yves Durand, Fabien Mitri, Yannick Knefati, Jean Vion-Dury

**Affiliations:** ^1^Center Hospitalier Intercommunal de Toulon La Seyne-sur-Mer, Toulon, France; ^2^Aix Marseille Univ, CNRS, PRISM, Marseille, France; ^3^Département Universitaire de Médecine Générale, Faculté des Sciences Médicales et Paramédicales, Marseille, France; ^4^Délégation à la Recherche Clinique, Centre Hospitalier Intercommunal de Toulon La Seyne-sur-Mer, Toulon, France; ^5^Pôle Universitaire de Psychiatrie, Assistance Publique-Hôpitaux de Marseille, Marseille, France

**Keywords:** anxiety, art-based intervention, ambulatory medicine, hemodialysis, schizophrenia

## Abstract

**Introduction:**

This study was conducted to determine the effectiveness of coloring activity of circular symmetrical shape with complex patterns, so-called mandala, on anxiety associated with chronic illness in three different ambulatory medical situations (general consultation, psychiatric day hospital, and hemodialysis session).

**Methods:**

Thirty patients were included in three groups and came from three different ambulatory medical situations: a hemodialysis group (*n* = 10), a psychiatric day hospital group (*n* = 10), and a nephrology consultation group (*n* = 10). We asked the patients to fill STAI-S and STAI-T questionnaires before to color complex circular shape with complex patterns, then to fill the STAI-S questionnaire again and a questionnaire on the experience of the activity.

**Results:**

The results show that the STAI-S score was significantly lower after coloring for the hemodialysis (*p* = 0.02) and psychiatric groups (*p* = 0.005) but not for the general consultation group (*p* = 0.26). STAI-T scores did not differ between groups. The distribution of colors in the mandala was different in the three groups of patients. A positive subjective experience of the activity was found in all groups.

**Discussion:**

These results show the effectiveness of a coloring activity of a circular shape with complex patterns on anxiety associated with chronic illness in care environment. The different distribution of the colors of the mandala in the three groups raises the question of the influence of the context on the mood of the patients and the deeper meaning of the choice of colors and their placement in the mandala. Our study reinforces the multiple applications of art activities in different medical disciplines and encourages their development within healthcare settings.

## 1 Introduction

The influence of art on health has been recognized for many years. Art is used as a therapy in many medical disciplines such as psychiatry, geriatrics, and oncology [National Collaborating Centre for Mental Health (UK), 2009; Puetz et al., 2013; Chancellor et al., 2014]. Research is also being carried out on the role of art in health promotion, prevention, and management of long-term chronic diseases (Camic, 2008; Haidet et al., 2016). The creative arts disciplines used in this context are mainly music, visual arts, movement-based creative expression, and writing-based expression. It has been shown that participation in artistic activities, either as an observer or as an initiator of own creative activities, can improve emotions, reduce anxiety, promote social relationships, and display an effect on various physiological parameters (Perruzza and Kinsella, 2010; Stuckey and Nobel, 2010).

Anxiety results in a psychological and physiological state characterized by a combination of physical, emotional, cognitive, and behavioral symptoms (Shah and Han, 2015). This state is accompanied by somatic signs of sympathetic nervous system hyperactivity such as heart palpitations, flushing, sweating, and tremors (Kahn and Fawcett, 2008). Anxiety can be physiological (as an adaptive process) or evolve into a pathological state, with a negative impact on daily activities. The evolution of pathological anxiety can be broken down into firstly a symptomatic stage, then a syndromic stage and at last the anxiety disorder (Clere and Robert, 2014). Patients with chronic illnesses have a higher risk of presenting mental disorders such as anxiety and depression as comorbidities (Health Quality Ontario, 2013).

Some studies show a high prevalence of anxiety in chronic hemodialysis patients (Birmelé et al., 2012). Hemodialysis, the most widely used treatment for chronic end-stage renal failure, is a technique for supplementing renal function and purifying blood through an extracorporeal circuit. The psychological impact of dialysis is multifactorial and related to the causative nephropathy, the nature of the procedure, itself, and the characteristics of the patient.

Anxiety is particularly common in patients with schizophrenia, reported in 65% of this group (Temmingh and Stein, 2015). Anxiety symptoms can present in various ways, as states secondary to delusions or hallucinations, or in reaction to one or more stressors (Braga et al., 2013). They increase the risk of schizophrenia relapse and are correlated the incidence of suicide attempts. They also worsen cognitive deficits, social stigma, functioning, and quality of life in schizophrenia (Llorca et al., 2014).

Beyond the associated chronic pathology, being in a healthcare environment and the physician’s presence can increase the level of anxiety. The physician and his office can be seen as a conditioned stimulus and can trigger anxiety (Gerin et al., 2006; van Bokhoven et al., 2009).

Coloring a complex shape has been shown to be effective in reducing anxiety (Curry and Kasser, 2005; Sandmire et al., 2012; van der Vennet and Serice, 2012). Curry and Kasser examined the effectiveness of coloring in reducing anxiety induced in students. In their study, students were divided into three coloring groups: one group colored a mandala, a second group colored a checkered geometric shape, and the last had a blank sheet of paper for free coloring. The results showed that mandala group and checkered shape group presented a significantly lower anxiety scores after coloring compared to the free form group. Mandala and checkered shape groups did not differ significantly from each other. In their discussion the authors attributed this similarity to the fact that the checkered shape was just as complex as the mandala and that structured coloring activity then serves to reduce anxiety (Curry and Kasser, 2005).

Van der Vennet and Serice replicated the Curry and Kasser’s study on students. The results showed that anxiety could be significantly reduced by coloring a mandala as opposed to subjects who colored a free form on a blank sheet of paper or a checkered shape. Thus, the results showed a difference between coloring the mandala and the checkered shape which differs from Curry and Kasser’s study. The final anxiety measure for the mandala group was significantly lower than the initial anxiety. The authors concluded that coloring and focusing on mandala reduces anxiety (van der Vennet and Serice, 2012).

The hypothesis of the effectiveness of coloring is that persons who color complex geometric shapes, have the opportunity to engage in an activity that moves them away from negative thoughts (Curry and Kasser, 2005). It is possible that coloring a complex predefined shape generates few creative thoughts and thus rather promotes a relaxed state, different from creating on a blank sheet of paper (Sandmire et al., 2012). This complex shape would allow one to focus the attention and to enter a state like meditation or hypnotic trance. Nevertheless, the studies by Curry and Kasser (2005), Sandmire et al. (2012) and van der Vennet and Serice (2012) did not analyze the colors used by participants on their coloring.

When coloring a complex shape, beyond the implicit or explicit cognitive processes (perception of shapes and colors, voluntary actions) unconscious processes (in the sense of psychoanalysis) might determine the choice of areas (or shapes) colored and the colors used. In fact, following Jung, we might think that complex symbolic processes are activated in many creative activities, including the use of archetypes (Jung et al., 1964).

So, in this work, we will pay attention to the areas colored and the colors used in each group of patients, making the hypothesis that the type of pathology could influence the underlying unconscious processes.

Based on these findings, we wanted to evaluate the effectiveness of coloring circular symmetrical shape with complex patterns (mandala) on state anxiety among chronically ill patients in three different ambulatory medical settings. We hypothesize that the choice of colors is important and may provide indirect information about consciousness configurations (including the unconscious processes) when coloring complex shape.

## 2 Materials and methods

### 2.1 Study design

The study was conducted between in the spring and summer of 2018 at the Toulon La-Seyne-sur-Mer Hospital. We studied the effect of coloring a complex shape in reducing anxiety in chronically ill patients in three different ambulatory medical settings: psychiatric day hospital (Psychiatry Group), hemodialysis service (Hemodialysis Group), and nephrology consultation service (Consultation Group). These three ambulatory situations are different. In the Psychiatry Group, patients come for regular therapeutic activities. In the Hemodialysis Group, patients come for regular care in the context of a chronic illness. In the Consultation Group, patients come for a consultation.

In their study Van der Vennet and Serice (van der Vennet and Serice, 2012), have compared the variation of the STAI-State score in an induced anxiety situation before and after the performance of a coloring of a complex shape. The mean of the reduction in the STAI-S score was 14.92 and the standard deviation was 11.14. For risk *α* = 0.05 and *β* = 0.20, we obtain a minimum number of nine subjects to include. We therefore decided to include 10 subjects per group.

**Figure 1:**
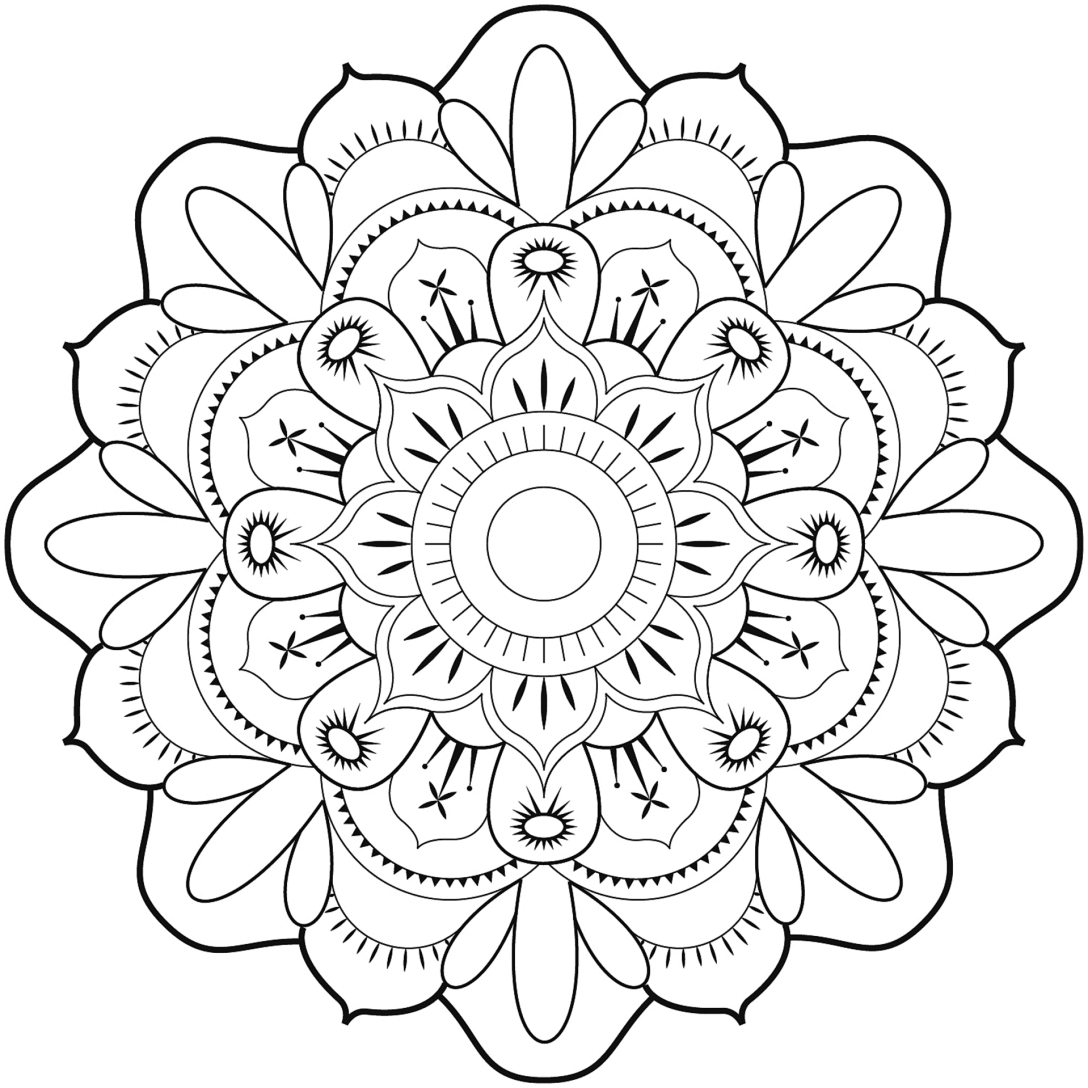
Complex shape (mandala) obtained and used for the study.

The complex shape (more commonly known as a mandala) was created using in vector format and then simplified so that it takes about 20 min to color entirely (in a healthy subject coloring at a normal rate). The resulting complex shape is shown in Figure 1. This shape contains 16 reference patterns repeated to obtain 108 areas that can be colored. The area of each reference pattern was calculated by a script. The distribution of colors according to the colored surface can be deduced by multiplying each area by the number of colored patterns of the same color. The number, the type of colored patterns can be counted, and the total surface area colored can be calculated.

**Figure 2:**
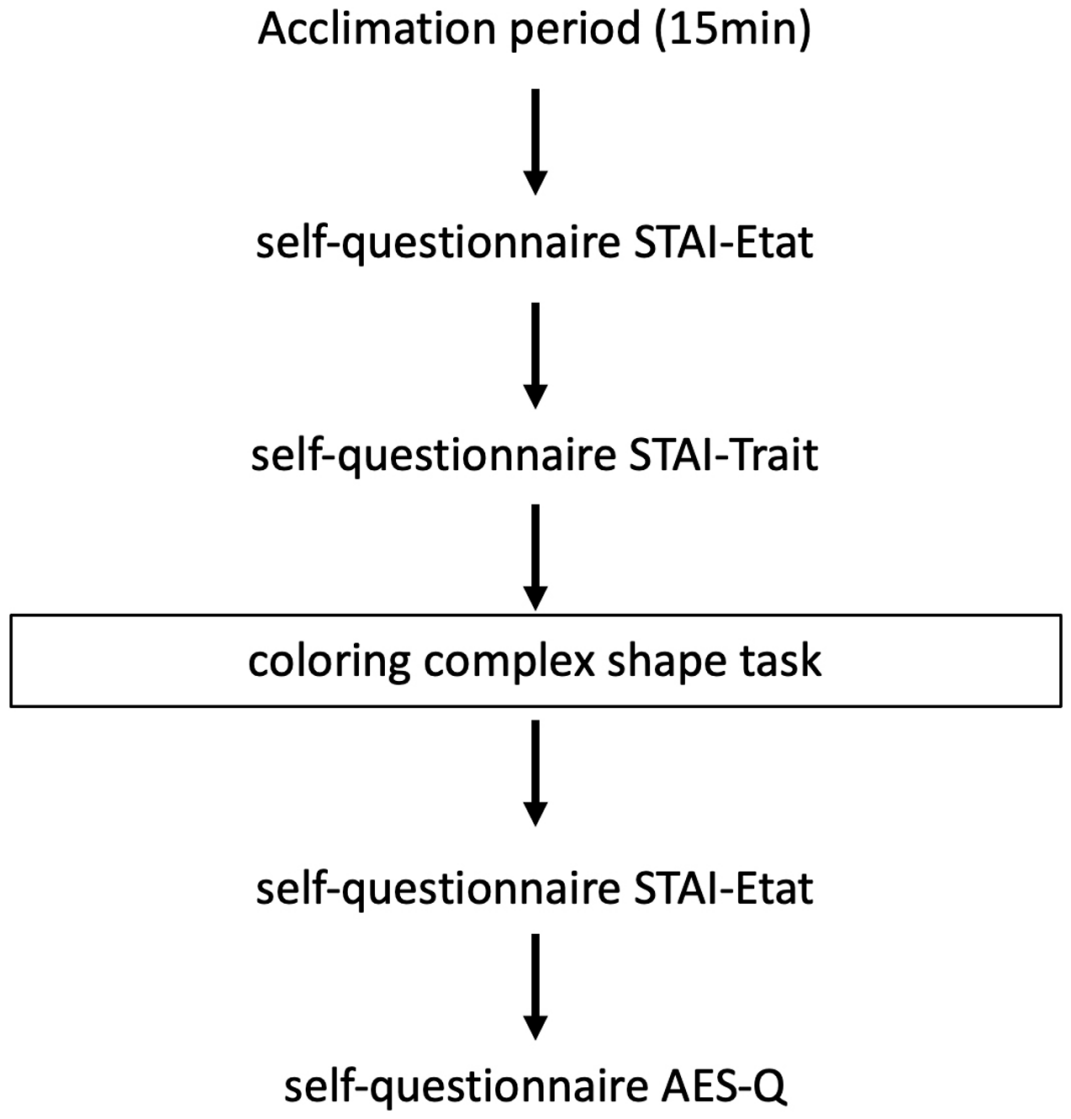
Overview of the study.

During a single visit as part of their usual follow-up and after obtaining their consent to the study, we asked patients in each group to complete the French version of the STAI-S and STAI-T self-questionnaires and then to complete the coloring of the mandala followed by the re-completion of the STAI-S self-questionnaire and the AES-Q questionnaire. A 15-min period (corresponding to the acclimation period) was allowed between patient arrival and questionnaire completion. The study course is shown in Figure 2.

The State Trait Anxiety Inventory (STAI) is a self-report measure of the presence and severity of current anxiety symptoms and a generalized propensity to be anxious (Spielberg, 1983; Julian, 2011). It is divided into two subscales. The Anxiety-State (STAI-S) scale which assesses the current state of anxiety and the Anxiety-Trait (STAI-T) scale which assesses the relatively stable aspects of a predisposition to anxiety (Groth-Marnat, 2009). The total score obtained from the scale is classified as follows: “no anxiety” (scores between 0 and 19), “mild anxiety” (scores between 20 and 39), “moderate anxiety” (scores between 40 and 59), “severe anxiety” (scores between 60 and 79), and “panic and crisis state” (scores between 80 and above) (Spielberg, 1983).

We developed a short questionnaire to get feedback from participants on this coloring task. It contains only five questions inspired by the ECTM2-Q questionnaire, and we have named it “Activity Evaluation and Satisfaction Questionnaire” (AES-Q). The *ECTM2-Q* questionnaire is a long questionnaire with 36 open-ended questions on participants’ experience of the activity (Rhondali et al., 2013; Laroque and Sudres, 2015; Sudres et al., 2016).

The complex shape was printed on a white A4 sheet. Colored pencils were given to patients. The colors available were black, brown, dark blue, light blue, purple, dark green, light green, orange, red, yellow, and pink. The patients were allowed to choose the colors that suited them. Patients could stop coloring whenever they wanted or decide not to finish the entire coloring. In the Psychiatry Group, the activity was carried out in an office made available for this study after having been proposed at the beginning of a day hospital. For the Hemodialysis Group, the activity was carried out during a hemodialysis session after having been proposed at the beginning of hemodialysis. For the Consultation Group, the activity was carried out in an office available for this study after being offered before the consultation.

### 2.2 Eligibility criteria

The inclusion criteria were the following for the three groups, patients aged over 18 and under 65, able to perform a coloring and answer a questionnaire. The Psychiatry group is corresponding to patients suffering from stabilized schizophrenia and admitted to a psychiatric day hospital for at least 10 consecutive days of care. The hemodialysis group is corresponding to patients having had at least six hemodialysis sessions with a valid dominant arm without arteriovenous fistula and currently undergoing dialysis treatment. The consultation group is corresponding to patients consulting a practitioner in hospital. Patients with advanced cognitive impairment impairing questionnaire response or coloring were excluded.

### 2.3 Ethical consideration

The work presented in this section was the subject of a clinical research protocol entitled COLORI whose promoter was the Center Hospitalier Intercommunal de Toulon La Seyne-sur-Mer. This protocol was submitted to and accepted by ethical committee (NCT03489980).

### 2.4 Evaluation of the data

Descriptive statistics were presented with means and standard deviations. After checking the distribution of data, the Wilcoxon test for paired series was used to compare the STAI-S score values before and after the intervention and the Kruskal-Wallis test was used to compare the STAI-T scores between the three groups. Significance was assessed as *p* < 0.05. The distribution of colors was calculated from the areas determined by the method described above and then analyzed descriptively. The pattern is fully colored if more than 85% of its surface has been colored. The colored area is then considered as the area of the pattern in question. Color symbolism was evaluated with *The Book of Symbols: Reflections on Archetypal Images. Cologne: Taschen; 2010*. All statistical analyses were performed with R software (R Development Core Team) (*R: A language and environment for statistical computing R Foundation for Statistical Computing, Vienna, Austria*).

## 3 Results

### 3.1 Population

Thirty-one patients were included in the study. One patient was withdrawn from the study because he did not color a sufficient area for a sufficient time. Of the remaining 30 patients, 10 patients (six males and four females) were included in Consultation group, 10 patients (seven males and three females) in Hemodialysis group, and 10 patients (eight males and two females) in Psychiatry group.

Of the three groups, the average age of the 30 subjects was 47 ± 2.51 years. Twenty-seven subjects were right-handed and three subjects were left-handed.

Patients included did not mention having undergone therapy for anxiety. The characteristics of the patients in each group are presented in Table 1.

**Table 1:** Characteristics of the subjects.

Group	*n*	Age (years)				Dominant hand		Coloring full	
		Mean	SD	Min	Max	Right-handed	Left-handed	*n*	%
Consultation	10	43.5	4.38	22	60	9	1	2	20%
Hemodialysis	10	53.5	4.23	22	64	8	2	7	70%
Psychiatry	10	42.2	4.03	20	55	10	0	4	40%
Total (three groups)	30	47	2.51	20	64	27	3	13	43.33%

### 3.2 Score STAI

before coloring were not different among the three groups (Kruskal-Wallis test, *p = 0*.58 and *p = 0*.19). Of the three groups, the STAI-S total score after coloring was significantly lower than the STAI-S score before coloring (Wilcoxon test, *p <* 0.005). The STAI-S total score after coloring was significantly lower than the STAI-S score before coloring in Hemodialysis group (Wilcoxon test, *p <* 0.05) and in Psychiatry group (Wilcoxon test, *p <* 0.05) but not in Consultation group (Wilcoxon test, *p =* 0.26) (Table 2).

**Table 2:** STAI-T and STAI-S score results by group.

Group	STAI-Trait		STAI-S^1^		STAI-S^2^		*p* value^3^
	Mean	SD	Mean	SD	Mean	SD	
Consultation	44.2	13.24	33.8	13.69	32.3	9.8	*p = 0.26*
Hemodialysis	40.1	14.39	31	8.18	25.9	4.33	*p = 0.02*
Psychiatry	42.2	9.15	39.2	10.25	32.3	9.71	*p = 0.0045*
Total (three groups)	42.2	12.1	34.66	2.11	30.17	8.62	*p = 0.00031*

^1^STAI-S before coloring; ^2^STAI-State after coloring; and ^3^Wilcoxon test for paired series between STAI-S before and after coloring.

### 3.3 AES-Q score

For the three groups combined, 50% (*n* = 15) of the subjects answered “rather yes” or “absolutely” to the question “Did you feel, notice a change and/or improvement in difficulties during and/or after this activity?”; 60% (*n* = 18) of the subjects in the three groups answered “rather yes” or “absolutely” to the question “Would you do an artistic activity again?” 76.67% (*n* = 23) of the subjects in the three groups answered “rather yes” or “absolutely” to the question “Did the activity improve your concentration?” 90% (*n* = 27) of the subjects in the three groups answered “rather yes” or “absolutely” to the question “Are you satisfied with this activity?” 70% (*n* = 21) of the subjects in the three groups answered “rather yes” or “absolutely” to the question “Did the activity make you forget about the care, the illness?” The results of the AES-Q questionnaire for each group are presented in Table 3.

**Table 3:** Responses to the AES-Q items according to questions.

AES-Q items	Number of responses				
	Not at all	Rather no	It depends on	Rather yes	Absolutely
Did you feel, notice a change and/or improvement in difficulties during and/or after this activity?	8 (26.27%)	5 (16.67%)	2 (6.67%)	12 (40%)	3 (10%)
Would you return to an artistic activity?	5 (16.67%)	3 (10%)	4 (13.33%)	12 (40%)	6 (20%)
Did the activity improve your concentration?	4 (13.33%)	1 (3.33%)	2 (6.67%)	18 (60%)	5 (16.67%)
Are you satisfied with this activity?	1 (3.33%)	0	2 (6.67%)	15 (50%)	12 (40%)
Has the activity made people “forget” the care, the illness?	5 (16.67%)	2 (6.67%)	2 (6.67%)	12 (40%)	9 (30%)

### 3.4 Colors

The averaged colored surface was 56% for Consultation group, 82% for Hemodialysis group, and 72% for Psychiatry group. In total, in the three groups, the colored surface was 70%. All colors were used in all groups. Pink, yellow, and red were the most used colors for Consultation group, light blue, pink, and yellow for Hemodialysis group and orange, red, and light blue for Psychiatry group (Table 4). In all groups, black was one of the least used colors.

**Table 4:** Percentage of the most represented colors in each group.

Color	Symbolic	% in Consultation group	% in Hemodialysis group	% in Psychiatry group
Red	Color of life (blood) and fire. Vitality but also danger. Libido (vital energy, sexuality), ardor, and daring. Transmutation of the ore and integration of the personality.	**8%**	**7%**	**11%**
Orange	Molten lava. Excitement. Physical body energy. Holding, guarding, and protecting. Energy, heat, and growth. Harvest. Transformation of the psyche.	**7%**	**8%**	**9%**
Yellow	Symbol of the highest things, joy, and fertility. Associated with gold. Also a symbol of betrayal and deception. Proud, ambitious, perceptive, and quick to anger. Increased energy and integration into life.	**7%**	**11%**	/
Pink	Emblem of femininity. The intense, deep color of pink is associated with seduction, tenderness, and romance. Pink is the color of love and sexuality. More commonly, pink symbolizes childhood.	**7%**	**7%**	**10%**
Blue	Unknown and depth of the sea (dark) or sky (light). Linked to eternity, to the highest. Cold, melancholy, isolation, spleen, and despair. Halfway between the black of despair and the white of hope.	/	**10% (light)**	**13% (light)**
				**7% (dark)**
Violet	Majesty, wealth, and honor. Symbolizes the union of opposites (red/blue) and the final union of substances. Process of spiritual growth and martyrdom.	/	**9%**	/
Green	Vibrant, vital energy. Creative power. Hope. But also an image of death and decay (decomposition, mold, pus, and mud).	/	**8%**	/

Only values greater than or equal to 7% were considered symbolically significant. The number in bold corresponds to the percentage of the shape’s colored surface filled for given color.

**Figure 3:**
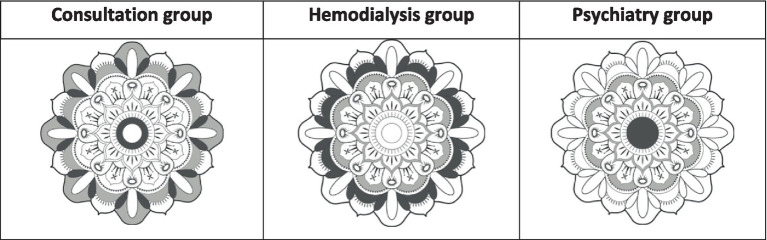
The two most colored patterns (in dark gray) and the least colored (in light gray).

The study of the percentages of filling of the mandalas of different forms according to the populations of patients makes it possible to put surprising differences (Figure 3).

The most colored patterns in the Consultation group is the periphery of the mandala. On the one hand, V-shaped colored structures suggest an opening toward the outside, or a welcome. In addition, these patients have colored only the periphery of the central circle of mandala. The least colored segments are those that would form an outer circle of protection. In Hemodialysis group, the most colored structures form an image of a rampart or barrier, with an empty center. Finally, in the Psychiatry group, the most colored structure is the central circle, as a central nucleus.

Not only do the general forms of coloring differ between populations, but the distribution of colors is also clearly different (Table 4).

In the Consultation group, the most represented colors (≥ 7%) are warm colors symbolic of energetic processes, heat and even eroticism. In this group, the percentages of colors (7.8%) are well balanced.

In the Psychiatry group, the most common color used was blue (20%), in both light and dark shades. Colors symbolizing transformations in the psyche (red, orange) are also well represented, as is pink, a color symbolizing sexuality (10%).

The most complex mandala color spectrum configuration comes from hemodialysis patients, with cold colors (green and blue represented) (Ronnberg and Martín, 2010).

## 4 Discussion

Our study was carried out to determine the effect of coloring mandala on state anxiety with chronic illness in three different ambulatory medical situations (psychiatric day hospital, hemodialysis session, and nephrology consultation).

The STAI-T score showed no significant difference between the three groups. The different patients generally present a comparable anxiety from a quantitative point of view, but probably not from a qualitative point of view. Future studies should also investigate the patients’ experience.

In the Psychiatry group, the STAI-S score was significantly reduced after coloring, meaning that anxiety decreased after the activity. Our results are in agreement with previously cited studies showing the benefits of art activities in patients with schizophrenia (Attard and Larkin, 2016). It has been suggested that the physical process of making art is considered to be calming and that it would allow distancing of symptoms in these patients (Attard and Larkin, 2016). Beyond the results obtained on anxiety, it would be interesting to know if this specific activity carried out on a regular basis makes it possible to reduce psychotic symptoms, positive or negative, and also quality of life, which has already been reported in certain studies (Attard and Larkin, 2016). This type of activity could also be extended to non-psychotic psychiatric disorders for which the benefits of art therapy have already been demonstrated (Uttley et al., 2015).

In Hemodialysis group, the STAI-S score was significantly reduced after coloring. The duration of activity is important to consider in this group of patients. We did not measure the duration of coloring but we found that the coloring time was relatively longer than in the consultation group. Indeed, the patient was free to color if he wished, and the duration of the hemodialysis sessions of about 4 h was suitable for this. It should be remembered that these sessions take place several times a week. Nishida and Strobino (2011) emphasized the possibility of using this time to carry out activities that are beneficial to the patient as opposed to passive activities. As Weldt showed, the patient who becomes active during the hemodialysis session regains confidence and forgets the hemodialysis machine (Cristina, 2003). This activity and creative arts activities in general could perhaps be developed to replace the classical passive occupations of patients in hemodialysis, such as watching television or using computer equipment. It should be noted that these artistic activities are easily achievable despite the extensive equipment of a hemodialysis ward and without interference with this treatment. The nurses on the ward noticed a positive impact when our protocol was carried out, which recalls the study by Ross et al. (2006). A greater attention and a benevolent attitude with a privileged patient-caregiver relationship resulted.

For the consultation group, the STAI-S score was not significantly reduced after coloring. The level of anxiety did not change after this artistic activity. We assume that our intervention was not adapted to this situation. Indeed, the time available for the activity was not the same as for the other groups. This may have led to frustration for the patient who did not take full advantage of the coloring activity depending on the waiting time. The time spent before the consultation was also prolonged for the subject who had to present earlier in order to participate in the study. More practical and flexible activities should be preferred in the waiting room.

For the AES-Q score, the overall results show a positive experience of coloring mandala for many of the patients who participated in the study. Most of the participants felt an improvement in their difficulties and were satisfied with this activity. They indicated that they would do it again.

Patients also overwhelmingly responded that the activity improved their concentration. All subjects in the Hemodialysis group responded in this way, which shows the focus of their attention. Indeed, a hemodialysis session is far from being devoid of intercurrent factors that often disturb patients (Ross et al., 2006). However, our activity enabled patients to forget or attenuate the presence of these obligatory events, such as machine alarms, nursing passes, blood pressure measurements, dressing care, or blood sampling.

One of the hypothesis of the effectiveness of coloring is that individuals who color complex geometric shapes, have the opportunity to engage in an activity that moves them away from negative thoughts (Curry and Kasser, 2005). This focus could generate a state of consciousness reminiscent of Csikszentmihalyi’s *Flow* (Nakamura and Csikszentmihalyi, 2014). *Flow* is defined as a rewarding state of deep involvement and absorption that individuals report when faced with a challenging activity and perceive adequate abilities to cope with these challenges. It is described as an optimal experience in which people are deeply motivated to persist in their activities (Peifer et al., 2022). Attention is said to be at the heart of this state of consciousness (Dietrich, 2004). The reduction of anxiety in this state is also thought to be related to avoidance (Jakobsson Støre and Jakobsson, 2022). It would also lead to a distortion of temporal experience, typically the impression that time is passing faster than normal (Nakamura and Csikszentmihalyi, 2014).

Csikszentmihalyi described this type of state during an artistic activity (Nakamura and Csikszentmihalyi, 2014). He found that the result of the creative production was less important to the creator than the process of making it. A directed and structured artistic activity could increase this state (Chilton, 2013). The use of coloring in a complex form would therefore be appropriate in this context. This focus on the activity would generate a state of *Flow* which would help patients to forget the illness and would have a beneficial effect on anxiety.

Nevertheless, we assume that the effect produced by the coloring of the complex shape is not only linked to the *Flow*. It seems associated with a change in the state of consciousness which can be compared to a light trance. Some authors state that coloring mandalas would have a meditative effect associated with this “Flow” state (Curry and Kasser, 2005; Mantzios and Giannou, 2018). Unlike to the studies of Curry and Kasser and Van der Vennet and Serice, we analyzed the distributions of colors. All colors were found in the groups and black was the color that was used the least. The use of specific multiple colors is important and could be related to the imagination of each patient in this meditative state during the coloring task. Csikszentmihalyi described a “trance-like” state that occurs during the process of art making (Csikszentmihalyi, 2013). We assume that this trance state would also be present with variable intensity depending on the patient. The different distribution of the colors of the mandala in the three groups might also depend on influence of the context (organization of waiting room, noises, and colors) on the patient’s mood. The combination of coloring forms and color symbolism seems to provide interesting information about the psychological processes at work in different populations.

The population of outpatients undergoing consultations express an openness and an internal energy that is probably linked to the fact that, overall, their pathologies have little effect on their vital impulse.

Patients suffering from psychiatric pathologies appear centered on themselves, but their vital and sexual energy (warm colors) seems preserved. However, mention should be made of the very high representation of blue (20%) in its two shades (dark and light). This may at least express the ambivalence found in schizophrenia.

Finally, the closure expressed by the coloring forms of hemodialysis patients could mean that they are putting themselves in a survival situation, separated from the world. The variety of colors, particularly the colder ones, probably reflects a difficulty in maintaining a vital impetus in the denial of death, illness and putrefaction, although hope is not absent from these processes.

Beyond the interest of the coloring and the light trance that it induces and which could explain the improvement of the scores of the STAI-S scale, the more detailed analysis of the coloring of the mandalas reveals unconscious processes whose expression is perhaps facilitated by this state of light trance. It seems to us indeed legitimate to think that the unconscious of the patients is expressed in the choice of the colors, since all the civilizations gave an often complex and sometimes contradictory symbolic direction to the colors. In a way, they are archetypes of the collective unconscious, if we look at C.G. Jung’s perspective.

Thus, several states of consciousness would follow during mandala coloring, suggesting a transformation of the structure of consciousness experience responsible for effect on anxiety. Further studies, using mixed method with phenomenological approach, are needed to explore these transformations of consciousness experience in this situation, with the aim of understanding both the feeling of anxiety and the experience of the choice of colors.

Our study has limitations. Like the studies by Curry and Kasser and van der Vennet and Serice, our samples in the groups are small (Curry and Kasser, 2005; van der Vennet and Serice, 2012). Our groups contain more men than women.

This is a preliminary study with a small sample size, and we have chosen not to include a control group. However, the results obtained encourage us to pursue this study with a randomized study including a control group. The protocol configuration did not allow measurement of coloring time.

Finally, duration of the diseases, associated treatments (especially psychiatric treatments) and emotional state outside of anxiety were not study, nor was the history of the patients included. Coloring time was also not measured. For future research, it will be important to have a larger number of patients in the groups and to compare them with a control group. Patients’ experience during the art activity should be studied qualitatively. The study of the long-term effect is also recommended (Koo et al., 2020).

## 5 Conclusion

We carried out studied whose objective was to evaluate the contribution of an artistic task such as coloring a mandala in the reduction of anxiety related to care, during three ambulatory medical situations. For Psychiatry and Hemodialysis groups, our study shows positive results with a decrease in their anxiety following the artistic activity. For Consultation group, we did not note any improvement or worsening of anxiety after coloring. A positive experience of the activity was shown for all participants. Although our study has limitations, it shows that an artistic activity such as coloring can be easily implemented in many outpatients’ medical situations and with a positive impact. Future work may be interested in refining the results and exploring the mechanism of effectiveness of coloring on anxiety.

## Data availability statement

The original contributions presented in the study are included in the article/supplementary material, further inquiries can be directed to the corresponding author.

## Ethics statement

The studies involving humans were approved by CPP OUEST III: 18.01.06/Id-RCB: 2017-A03537-49. The studies were conducted in accordance with the local legislation and institutional requirements. The participants provided their written informed consent to participate in this study.

## Author contributions

MD: Writing – original draft. EO: Writing – original draft. J-YD: Writing – original draft. FM: Writing – original draft. YK: Writing – original draft. JV-D: Writing – original draft.
